# The α7 nicotinic acetylcholine receptor agonist PNU-282987 ameliorates sepsis-induced acute kidney injury through CD4^+^CD25^+^ regulatory T cells in rats

**DOI:** 10.17305/bjbms.2022.7111

**Published:** 2022-04-10

**Authors:** Xiaocui Shi, Juncong Li, Yuzhen Han, Jingyi Wang, Qingping Li, Yue Zheng, Wenxiong Li

**Affiliations:** Department of Surgical Intensive Critical Unit, Beijing Chao-yang Hospital, Capital Medical University, Beijing, China

**Keywords:** PNU-282987, sepsis, sepsis-induced acute kidney injury, acute kidney injury, α7 nicotinic acetylcholine receptor, Tregs

## Abstract

The ameliorative effects of α7 nicotinic acetylcholine receptor (α7nAChR) agonists have been demonstrated in acute kidney injury (AKI) caused by multiple stimulations. However, the ameliorative effect of α7nAChR on sepsis-induced AKI (SAKI) in the cecal ligation and puncture (CLP) model is unclear. The previous studies have demonstrated that α7nAChR is highly expressed on the surface of CD4^+^CD25^+^ regulatory T cells (Tregs). However, the role of Tregs in SAKI is unclear. We hypothesized that Tregs might play a role in the ameliorative effect of α7nAChR on SAKI. Hence, in this study, we determined the effects of PNU-282987 (a selective α7nAchR agonist) on SAKI and evaluated whether PNU-282987 would attenuate SAKI through regulating Tregs. Our study showed that immediate administration of PNU-282987 after CLP surgery in rats improved renal function, reduced levels of systemic inflammatory factors (tumor necrosis factor-α, interleukin-6, etc.), inflammatory cell infiltration and tubular apoptosis in renal tissues, and increased forkhead/winged helix transcription factor p3 (Foxp3) and cytotoxic T-lymphocyte-associated protein 4 (CTLA-4) expression indicating activated Tregs. Moreover, in *in vitro* experiments, isolated Tregs cocultured with PNU-282987 also displayed enhanced expression of CTLA-4 and Foxp3. Furthermore, Tregs were cocultured with PNU-282987 for 24 hours and then reinfused into rats through the tail vein immediately after CLP surgery, and a significant renal protective effect was observed 24 hours postoperatively. These results demonstrate that PNU-282987 exerts its renal protective effects on SAKI through activation of Tregs.

## INTRODUCTION

Sepsis or septic shock is a leading cause of death in intensive care patients. Sepsis has a particularly high incidence and mortality rate in recent years, responsible for 50% or more of acute kidney injury (AKI) cases in the intensive care unit (ICU) [1]. AKI occurs in up to 60% of septic patients in ICU, with sepsis being the most prevalent trigger [2-4]. Sepsis-induced AKI (SAKI) is a complex syndrome involving multiple physiological alterations, including renal hemodynamic changes, immune cell activation, massive release of inflammatory molecules, and endocrine dysregulation [5]. Moreover, sepsis can further damage the kidney by increasing the systemic load of inflammatory mediators, further promoting the development of AKI [6]. However, the clinical efforts to alleviate the development of SAKI have not been successful [7]. Therefore, it has been urgent to understand the mechanisms of SAKI and develop clinical strategies that could alleviate SAKI development.

Immune homeostasis in the organism is mainly determined by the balance between pro-inflammatory and anti-inflammatory T-helper lymphocyte subsets [8]. CD4^+^CD25^+^ regulatory T cells (Tregs) are the primary regulators of immunosuppression [9]. Tregs inhibit the immune response mediated by other immune cells, such as T cells, and play an important role in maintaining the body’s immune balance and preventing autoimmune diseases and transplant rejection [10]. Tregs suppress the inflammatory response by secreting anti-inflammatory cytokines which can act directly on effector cells [11]. Some studies have shown that Tregs could not only prevent the occurrence but also promote the recovery of renal ischemia-reperfusion injury (IRI) [12,13]. Adoptive transfer of isolated Tregs before ischemia markedly protects mice against renal dysfunction and tissue injury [13,14]. *In vivo* experiments in mice also showed that increased Tregs would prevent IRI-AKI tubular injury and protect the kidney from developing fibrosis [15,16]. An observational study showed that the level of serum soluble CD25, a marker of Tregs, was significantly elevated in patients with SAKI [17]. However, the exact role of Tregs in SAKI remains unclear.

The α7 nicotinic acetylcholine receptor (α7nAChR) has been identified as a unique receptor of the cholinergic anti-inflammatory pathway, which exerts anti-inflammatory effects in a variety of diseases [18]. Moreover, α7nAchR is extensively expressed in multiple immune cells, such as macrophages, T cells, and B cells [19]. Agonists of α7nAchR have a protective role against inflammatory conditions, such as kidney IRI and acute lung injury, and can effectively reduce the levels of related inflammatory cytokines [20,21]. PNU-282987 is a selective agonist of α7nAchR with high affinity, which can alleviate AKI induced by lipopolysaccharide (LPS), and significantly downregulate systemic levels of tumor necrosis factor-α (TNF-α), interleukin (IL), and IL-1β [22]. Experiments in mice have shown that α7nAchR is expressed on the surface of Tregs, and PNU-282987 can enhance the immunosuppressive ability of Tregs [23]. Thus, we hypothesized that PNU-282987 could ameliorate SAKI by regulating the function of Tregs. This study aimed to validate this hypothesis by establishing the SAKI rat model and applying the adoptive transfer of Tregs cocultured with PNU-282987.

## MATERIALS AND METHODS

### Animal model

A 6-8week-old male Sprague Dawley rats weighing 200-220 g (Vital River Laboratories, Beijing, China) were provided with free access to water and food for a 72 h acclimation period before being used for experiments. Rats were housed in a room that maintained constant temperature and humidity and were subjected to a 12 hours light/dark cycle. The animal studies were approved by Capital Medical University Animal Care and Use Committee (ethics number P2020-3-17-54). Before all surgical procedures, rats were anesthetized with isoflurane by inhalation (induction at 5%, 2 L/minutes and thereafter at 3%, 0.7 L/minutes) [24]. SAKI was induced by cecal ligation and puncture (CLP) in rats. The detailed procedures were as follows: The abdomen was disinfected and a 2-3 cm longitudinal skin midline incision was made; then, the cecum in the abdominal cavity was identified and the mesentery from the distal end of the large intestine was separated carefully to avoid bruising the mesenteric vessels; next, the contents of the cecum toward its distal end were squeezed gently and the distal half of the cecum with a 3.0 silk thread was ligated tightly; next, a sterile 16-gauge needle was used to puncture through-and-through twice, and then a small amount of feces was squeezed lightly; the cecum was then put back and the abdominal cavity was closed by sutures layer by layer [25]; then, rats were resuscitated by subcutaneously injecting prewarmed (37°C) normal saline (0.9%) (5 ml per 100 g body weight), and rats were put back in cages.

### Experimental protocols

To determine whether PNU-282987 can activate Tregs and improve SAKI, the rats were randomly divided into four groups, that is, the sham, CLP, CLP+PNU-low, and CLP+PNU-high groups, with eight rats per group. Surgical procedures, including anesthesia, laparotomy, separation of the distal cecum from the mesangium of the large intestine, and wound closure, were performed on rats in the sham group. The above-described standard surgical procedures were performed on rats in the CLP group. Other than standard surgical procedures, the rats in the CLP+PNU-low and CLP+PNU-high groups were also intraperitoneally injected with PNU-2828987 (Abmole Bioscience Inc, Houston, USA) at low (0.5mg/kg body weight) and high (1 mg/kg body weight) doses, respectively, immediately after the CLP surgery [26]. All rats were placed in metabolic cages for 24 hours to collect urine. Then, 1-3 ml of blood was also collected from the heart of the rats after anesthesia with 5% isoflurane. After blood collection, the rats were sacrificed and intact kidney and spleen tissues were collected immediately under aseptic operations.

To further verify whether PNU-282987 improves SAKI by regulating Tregs, we also performed adoptive Treg transfer experiments. Briefly, CD4^+^CD25^+^ Tregs were extracted from the spleen of normal rats and cultured in normal culture media (FBS + RPMI 1640) containing PNU-282987 at concentrations of 0 mol/L, 0.5 mol/L, and 1 mol/L for 24 hours, corresponding to the Tregs, Tregs+PNU-low, and Tregs+PNU-high groups, respectively. After coculture for 24 hours, the culture supernatant was collected and 5 × 10^5^ Tregs in each group were used for staining and flow cytometric analysis. Moreover, 2 × 10^6^ Tregs were injected into the rats in the Tregs, Tregs+PNU-low, and Tregs+PNU-high groups through the tail vein immediately after CLP surgery. For rats in the control group (the Tregs group), phosphate buffer saline (PBS) was injected through the tail vein instead. Accordingly, the four groups were named the CLP+PBS group, CLP+Tregs group, CLP+Tregs (PNU-low) group, and CLP+Tregs (PNU-high) group. Eight rats were used in each group. All rats were placed in metabolic cages for 24 hours to collect urine for 24 hours. Then, 1-3 ml of blood was collected from the heart of rats after anesthesia with 5% isoflurane. After blood collection, the rats were euthanized under isoflurane-induced anesthesia and sacrificed, and intact kidney and spleen tissues were collected immediately under aseptic conditions.

### Histopathological evaluation

After washing with PBS, the kidney tissues were fixed in 4% paraformaldehyde for 24 hours, followed by embedding in paraffin. The blocks were then sliced into 4 mm segments and transferred to glass slides, which were stained with hematoxylin and eosin (H&E) for histopathological evaluation. A white light microscope (Leica Microsystems GmbH, Wetzlar, Germany) was used to observe and photograph the stained sections. Histopathological evaluation of the stained sections was performed using the kidney histological damage score. Specifically, for each specimen, at least five random fields at ×200 magnification were scored. The following aspects, including epithelial cell vacuolization and degeneration, tubular cell flattening, hyaline cast, tubular dilatation, and debris materials in the tubular lumen, were scored, with each aspect having a maximal 1 point and a minimal 0 point. A higher total score indicates more severe damage (the maximum score is 5, while a score of 0 is assigned to normal tissue without damage) [27,28].

### TUNEL staining

Apoptosis of tubular epithelial cells was assessed by the terminal deoxynucleotidyl transferase-mediated (TdT) dUTP nick-end labeling (TUNEL) assay [29] using an *in situ* apoptosis detection kit (Beyotime Biotechnology, Beijing, China), according to the manufacturer’s instruction. TUNEL-positive cells were counted in ten random fields at ×200 magnification [30].

### Biochemical and enzyme-linked immunosorbent assay (ELISA) analyses

Plasma samples were analyzed with a serum creatinine (Scr) assay kit (Nanjing Jiancheng Bioengineering Institute, Nanjing, China) and a blood urea nitrogen (BUN) assay kit (Nanjing Jiancheng Bioengineering Institute, Nanjing, China) 24 hours after the CLP surgery. Plasma TNF-α, IL-6 and urinary neutrophil gelatinase-associated lipocalin (NGAL), kidney injury molecule 1 (KIM-1), transforming growth factor-1 β (TGF-β1), and IL-10 were analyzed using a commercial ELISA kit (Beijing 4A Biotech Co. Ltd, Beijing, China).

### Flow cytometry

First, spleen single-cell suspensions in rats of different groups were prepared. CD4^+^CD25^+^ Tregs were enriched with positive selection using the Miltenyi Biotec magnetic sorter (Miltenyi Biotec, Germany) according to the manufacturer’s instruction. Then, the collected Tregs were stained with PE-conjugated anti-cytotoxic T-lymphocyte-associated protein 4 (CTLA-4) and PE-cy7-conjugated anti-Foxp3 antibodies (eBiosciences, CA, USA). Then, the mean fluorescence intensity (MFI) of Foxp3 (a marker of Treg cells) and CTLA-4 (a negative regulatory receptor on Tregs) was recorded by flow cytometry using the BD FACSCalibur System (BD Bioscience, USA). Flow cytometric data were analyzed using the FlowJo V10 software (Treestar Software, USA).

### Adoptive transfer of Tregs

Tregs were sorted out from normal rat spleens and cultured in media containing different concentrations of PNU-282987 for 24 h. Then, 2 × 10^6^ Tregs were infused into each rat immediately after CLP surgery through tail vein injection. For rats in the control group, an equal volume of PBS was injected instead.

### Western blotting

The collected kidney tissues were grounded into powder in liquid nitrogen, which was then lysed in RIPA lysis buffer (Beyotime, Shanghai, China) at 4°C for 20 minutes. For sorted Tregs, RIPA lysis buffer was added to the cells directly. The samples were centrifuged at 12,000 rpm/minutes at 4°C for 30 minutes. The supernatant was collected and protein concentration was determined using a bicinchoninic acid kit. Proteins (50 μg per lane) were separated using 12% sodium dodecyl sulfate-polyacrylamide gel electrophoresis. The proteins were then electrotransferred to polyvinylidene fluoride (PVDF) membranes (Merck KGaA, Darmstadt, Germany). After blocking for 1 hours in Rapid Blocking Solution (Beyotime, Shanghai, China), the PVDF membrane was incubated with the primary antibodies, that is, the AChR alpha 7 antibody (GeneTex, CA, USA) (1:1000) and GADPH antibody (Beyotime, Shanghai, China) (1:2000), overnight at 4°C. After washing with Tris-buffered saline containing Triton X-100, the membrane was incubated with the horseradish peroxidase-conjugated secondary antibody (Beyotime, Shanghai, China) (1:2000) for 1 hours at room temperature, and then a chemiluminescence kit (Beyotime, Shanghai, China) was used to detect protein bands.

### Immunofluorescence staining

Sorted CD4^+^CD25^+^ Tregs were washed with PBS, fixed with 4% paraformaldehyde at room temperature for 30 minutes, and centrifuged. After washing with PBS, the cells were resuspended and blocked in 1% BSA in TBST at room temperature for 30 minutes, followed by incubation with rabbit anti-α7nAChR polyclonal antibody (GeneTex, CA, USA) at 4°C overnight. After washing with PBS and centrifuge, the cells were incubated with fluorescein isothiocyanate -labeled goat anti-rabbit polyclonal antibody (Beyotime, Shanghai, China) (1:100) at 4°C overnight. After washing with PBS, the stained cells were resuspended in PBS and dropped onto 24-well plates, followed by observation under the confocal fluorescence microscope.

### Statistical analysis

All the data were analyzed using the SPSS 26.0 software (SPSS Inc., Chicago, IL, USA) and were expressed as mean ± standard deviation (SD). Student’s *t*-test and non-parametric test (Mann–Whitney U-test) were used for comparisons between the two groups. ANOVA followed by Bonferroni correction or non-parametric test (Mann–Whitney U-test) was used for comparisons of multiple. A value of *p* < 0.05 was considered statistically significant. Graphs were generated with the GraphPad Prism version 8.1.2 software (GraphPad Software, CA, USA).

## RESULTS

### Establishment of a SAKI rat model

**FIGURE 1:**
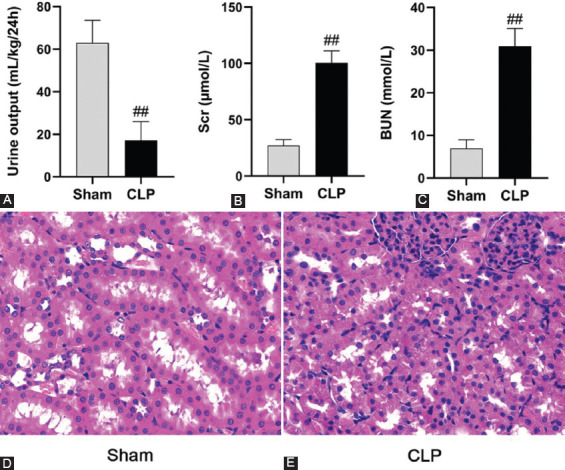
Establishment of sepsis-induced acute kidney injury model in rats. (A-C) The decremental urine output and increased serum creatinine and blood urea nitrogen were observed in the cecal ligation and puncture (CLP) group compared to the sham group; (D) representative H&E-stained sections (original magnification, ×400) of the rat kidney were obtained from the sham group 24 hours after surgery; and (E) Representative H&E-stained sections (original magnification, ×400) of the rat kidney were obtained from the CLP group 24 hours after surgery. The values are expressed as the mean ± SD (n = 8 in each group). ^##^*p* < 0.01 versus the sham group.

Compared to the sham group, the rats in the CLP group showed features such as lethargy, body crouching, inactive eating and drinking, a sluggish responsiveness to the outside environment, an increased respiratory rate, bloody secretions around the eyes, coarse and less glossy hair, and loose feces. All rats were euthanized 24 hours after surgery and the plasma, urine, and kidney tissues were collected. Then, the rats were further examined immediately. Laparotomy revealed foul-smelling bloody exudate, intestinal edema and adhesion, necrosis, and blackness of the cecum in the CLP group. Compared to the sham group, the urine volume in the CLP group was significantly lower (*p* < 0.01) (Figure 1A), and the levels of Scr and BUN were significantly higher in the CLP group (*p* < 0.01) (Figure 1B and C). No obvious pathological changes were observed in the renal histopathological sections of the sham group (Figure 1D). However, the renal histopathological sections of the CLP group revealed vacuolization of renal tubular epithelial cells, infiltration of renal interstitial inflammatory cells, and exfoliated epithelial cells and fragments in the renal tubular lumen (Figure 1E). Thus, an SAKI rat model was successfully established using the CLP surgery.

### Expression of α7nAchR on the surface of rat CD4^+^CD25^+^ Tregs

**FIGURE 2:**
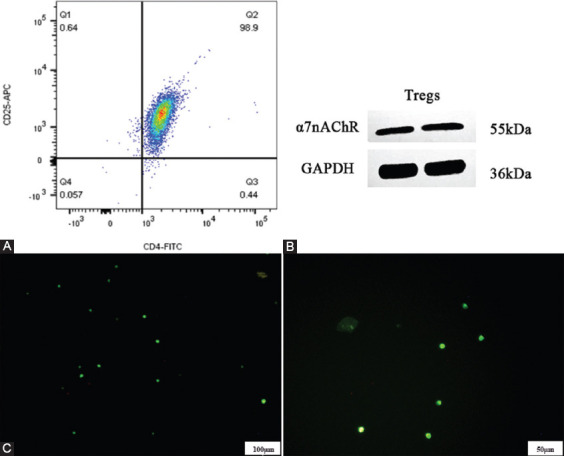
Expression of a7 nicotinic acetylcholine receptor on the surface of rat CD4+CD25+ regulatory T Cells. (A) The purity of CD4+CD25+Tregs cells obtained after the single cells of normal rat spleen were sorted by magnetic beads was above 90%; (B) the a7nAChR band of Treg cells was clearly detected in Western blotting; and (C) After confocal fluorescence microscopy observation, Tregs cells emit green fluorescence under laser excitation.

CD4^+^CD25^+^ regs were isolated from normal spleens of SD rats using a positive selection kit. The purity of isolated Tregs was analyzed by flow cytometry (Figure 2A). When the purity is above 90%, the purified Tregs can be used for subsequent experiments. The expression of α7nAchR in CD4^+^CD25^+^ Tregs was detected by Western blot. As shown in Figure 2B, the α7nAChR band was clearly detected on the PVDF membrane, indicating that α7nAChR is expressed in rat Tregs. We also carried out immunofluorescence staining against α7nAChR. As shown in Figure 2C, green fluorescence representing α7nAChR signal was observed on the cellular surface, further indicating α7nAChR is expressed on rat Tregs.

### Effects of PNU-282987 on indicators of rat kidney injury 24 hours after the CLP surgery

**FIGURE 3:**
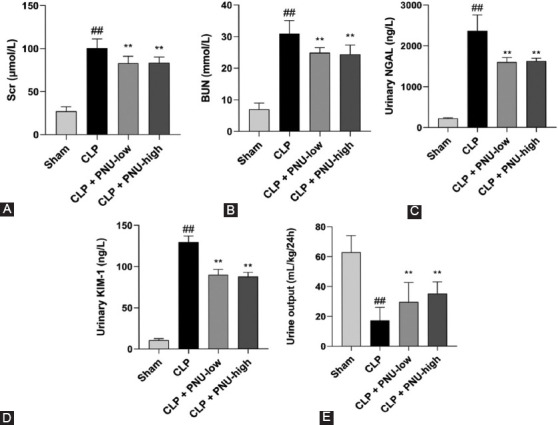
PNU-282927 reduced the indicators of kidney injury 24 hours after cecal ligation and puncture (CLP) surgery in rats. (A-E) The levels of serum creatinine, blood urea nitrogen, urinary neutrophil gelatinase-associated lipocalin, kidney injury molecule 1, and urine output are shown in the four groups, respectively. The values are expressed as the mean ± SD (n = 8 in each group). ^##^*p* < 0.01 versus the sham group; **p < 0.01 versus the CLP group.

The levels of Scr, BUN and urinary NGAL, KIM-1 in the CLP + PNU-low, and CLP + PNU-high groups were moderately, but significantly lower than those in the CLP group 24 hours after the CLP surgery, respectively (Table S1). However, between the CLP+PNU-low and CLP+PNU-high groups, no significant difference in levels of these markers was observed (Figure 3A-D). The urine volumes of rats in the CLP+PNU-low and CLP+PNU-high groups were significantly higher than that in the CLP group 24 hours after the CLP surgery (*p* < 0.01) (Figure 3E).

### Effects of PNU-282987 on rat renal histopathology and apoptosis 24 hours after the CLP surgery

**FIGURE 4:**
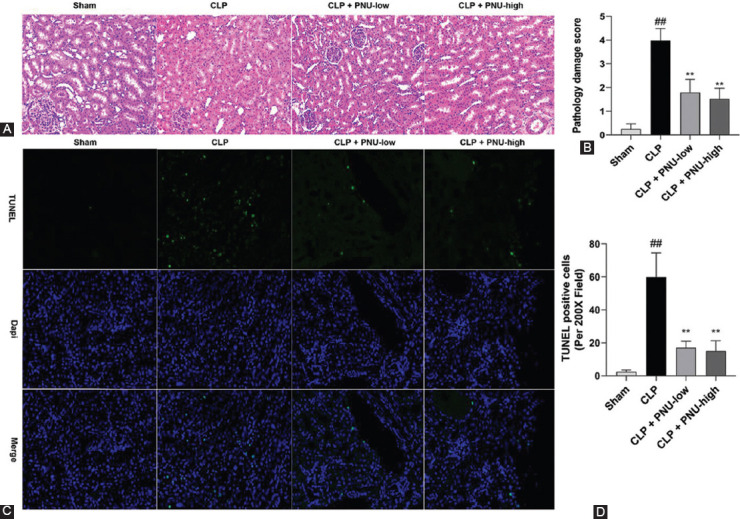
PNU-282987 ameliorated renal histopathology and apoptosis 24 hours after cecal ligation and puncture (CLP) surgery in rats. (A) The representative images of H&E stained kidney sections (original magnification, ×200) in the sham, CLP, CLP + PNU-low, CLP + PNU-high groups; (B) the kidney histological damage scores from the four groups; (C) tubular apoptosis (TUNEL staining) and representative photomicrographs of rats (original magnification, ×200); and (D) apoptosis by counting the number of TUNEL positive cells per high power field using random sections and the mean apoptosis scores. The values are expressed as the mean ± SD (n = 8 in each group). ^##^*p* < 0.01 versus the sham group; **p < 0.01 versus the CLP group.

H&E staining was used to evaluate renal histological changes. As shown in Figure 4, the kidney histological damage scores were significantly higher in the CLP group compared to the sham group, and significantly lower in the CLP+PNU-low and CLP+PNU-high groups compared to the CLP group. However, no significant difference was observed between the CLP+PNU-low and CLP+PNU-high groups (Table S1). Representative images for H&E staining for each group are shown in Figure 4A and the kidney histological damage scores of each group are shown in Figure 4B. Moreover, the number of TUNEL positive cells per high power field was significantly lower in the CLP+PNU-low and CLP+PNU-high groups, while no significant difference was observed between the CLP+PNU-low and CLP+PNU-high groups (Figure 4C and D).

### Effects of PNU-282987 on systemic inflammation in rats 24 hours after the CLP surgery

**FIGURE 5:**
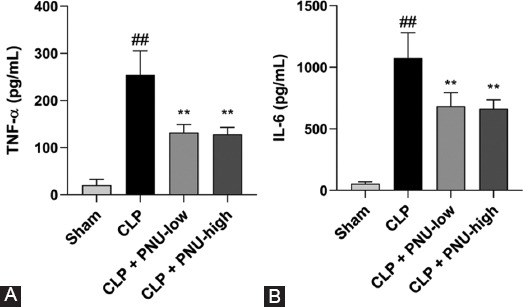
PNU-282987 ameliorated systemic inflammation 24 hours after cecal ligation and puncture (CLP) surgery in rats. (A) Serum TNF-α levels are shown in the four groups and (B) IL-6 levels are shown in the four groups. The values are expressed as the mean ± SD (n = 8 in each group). ^##^*p* <0.01 versus the sham group; **p < 0.01 versus the CLP group.

The circulating TNF-α and IL-6 levels were significantly higher in the CLP group compared to the sham group (Figure 5) and were significantly lower in the CLP+PNU-low and CLP+PNU-high groups compared to the CLP group. However, no significant difference was observed between the CLP+PNU-low and CLP+PNU-high groups.

### Effects of PNU-282987 on the expression of CTLA-4, Foxp3 and α7nAChR, and the production of TGF-β1 and IL-10 in Tregs of SAKI rats

**FIGURE 6:**
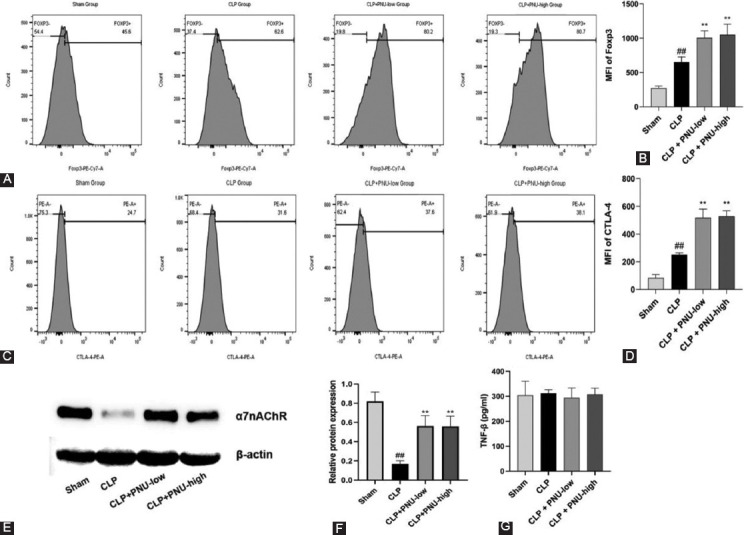
PNU-282987 increased expressions of Foxp3, CTLA-4, α7nAChR, and the production of TGF-β and IL-10 in Tregs of sepsis-induced acute kidney injury rats. (A) Representative flow cytometry images of Foxp3 on Tregs are shown in the four groups; (B) the MFI of Foxp3 on Tregs are shown in the four groups; (C) representative flow cytometry images of CTLA-4 on Tregs are shown in the four groups; (D) representative MFI of CTLA-4 on Tregs is shown in the four groups; (E) the a7nAChR band of Treg cells was clearly detected in Western Blotting; (F) the relative expression of α7nAChR was normalized to β-actin; and (G) the production of TGF-β1 was determined by ELISA. The values are expressed as the mean ± SD (n = 8 in each group). ^##^p < 0.01 versus the sham group; **p < 0.01 versus the CLP group.

In *in vivo* experiment, the expression levels of Foxp3 and CTLA-4 in Tregs 24 hours after surgery were significantly higher in the CLP group compared to the sham group and significantly higher in the CLP+PNU-low and CLP+PNU-high groups compared to the CLP group. Representative flow cytometry images and MFI of Foxp3 and CTLA-4 on Tregs in the four groups are shown in Figure 6A-D, respectively. Compared with the sham group, expression levels of α7nAChR were significantly reduced in the CLP group, highlighting the role of CLP surgery in this process. Compared with the CLP group, α7nAChR was significantly upregulated in the CLP+PNU-low and CLP+PNU-high groups, indicating the role of PNU-282987 treatment in regulating α7nAChR expression in Tregs (Figure 6E and F). However, no significant difference was observed between the CLP+PNU-low and CLP+PNU-high groups. Further, levels of IL-10 were not detected and levels of TGF-β1 were not significantly different in culture supernatants of the four groups (Figure 6G).

### Effects of PNU-282987 on the expressions of Foxp3 and CTLA-4 and the production of TGF-β1 and IL-10 in Tregs *in vitro*

**FIGURE 7:**
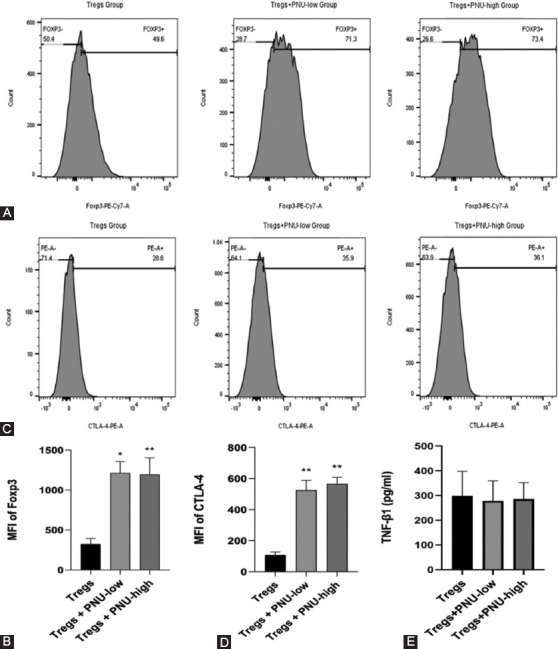
PNU-282987 increased expressions of Foxp3, CTLA-4 in Tregs *in vitro*, and the effects of PNU-282987 on production of TGF-β1 and IL-10 in Tregs *in vitro*. (A) Representative flow cytometry images of Foxp3 on Tregs are shown in the three groups; (B) the mean fluorescence intensity (MFI) of Foxp3 in Tregs is shown in the three groups; (C) representative flow cytometry images of CTLA-4 in Tregs are shown in the three groups; (D) representative MFI of CTLA-4 in Tregs is shown in the three groups; and (E) the production of TGF-β1 was determined by ELISA. The values are expressed as the mean ± SD (n=8 in each group). ^#^*p* < 0.05 versus the Tregs group; ^##^*p* < 0.01 versus the Tregs group.

The expression levels of Foxp3 and CTLA-4 in Tregs were significantly higher in the Tregs+PNU-low group and Tregs+PNU-high groups compared to the Tregs group (Table S2). Representative flow cytometry images and MFI of Foxp3 and CTLA-4 on Tregs in the three groups are shown in Figure 7A-D, respectively. Further, levels of IL-10 were not detected and levels of TGF-β1 were not significant in culture supernatants of the three groups (Figure 7E).

### Adoptive transfer of Tregs treated with PNU-282987 ameliorated SAKI in rats

**FIGURE 8:**
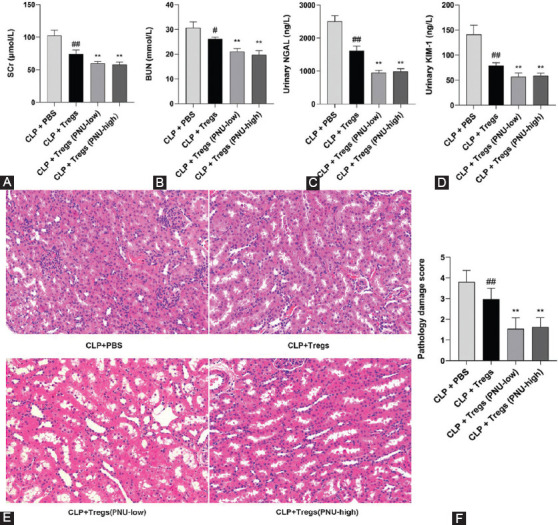
Adoptive transfer of CD4^+^CD25^+^ regulatory T cells (Tregs) incubated in media containing PNU-282987 ameliorated kidney injury 24 hours after the cecal ligation and puncture (CLP) surgery in rats. (A-D) The levels of serum creatinine, blood urea nitrogen, urinary neutrophil gelatinase-associated lipocalin, and kidney injury molecule 1 are shown in the four groups; (E) representative images of the pathology (×200) are shown in the four groups; and (F) the kidney histological damage scores are shown in the four groups. The values are expressed as the mean ± SD (n = 8 in each group). ^#^*p* < 0.05 versus the CLP + PBS group; ^##^*p* < 0.01 versus the CLP + PBS group; **p < 0.01 versus the CLP + Tregs group.

Scr, BUN, NGAL, and KIM-1 were used as biomarkers of AKI 24 hours after CLP surgery. Tregs cocultured with PNU-282987 for 24 hours or not cocultured with PNU-282987 were infused into the rats through tail vein injection. As shown in Figure 8A-D, levels of the four biomarkers were significantly lower in the CLP+Tregs group compared to the CLP+PBS group, and significantly lower in the CLP+Tregs (PNU-low) and CLP+Tregs (PNU-high) groups compared to the CLP+Tregs group. The kidney histological damage scores were significantly lower in the CLP+Tregs group compared to the CLP+PBS group (*p* < 0.01), and significantly lower in the CLP+Tregs (PNU-low) and CLP+Tregs (PNU-high) groups compared to the CLP+Tregs group (*p* < 0.01) (Figure 8F). Representative images of histological damage in the CLP+PBS, CLP+Tregs, CLP+Tregs (PNU-low), and CLP+Tregs (PNU-high) groups are shown in Figure 8E. The biomarkers of early acute kidney injury and pathology damage score after the adoptive transfer of Tregs in the SAKI rats are shown in Table S3]

## DISCUSSION

The α7nAChR agonists have been shown to improve ischemia-reperfusion kidney injury, cisplatin-related, and LPS-related AKI [22,31,32], but have not been shown to improve AKI in the CLP model, which displays symptoms consistent with SAKI [33]. In the present study, *in vivo* experiment showed that administration of PNU-282987 improved renal function, reduced apoptosis in tubular cells, increased expression of CTLA-4 and Foxp3, and thus activated Tregs in the CLP rats. In *in vitro* experiments, the expression levels of CTLA-4 and Foxp3 in Tregs cocultured with low or high concentrations of PNU-282987 were significantly higher compared with that in untreated Tregs. Furthermore, obvious renal protective effects were observed after the adoptive transfer of isolated Tregs that were cocultured with low or high concentrations of PNU-282987 in the CLP rats. Thus, we have demonstrated that PNU-282987 exerts renal protective effects on SAKI through activating Tregs.

SAKI occurs primarily as a result of infection-induced renal dysfunction, and in early sepsis, the kidney injury is mainly caused by an excessive inflammatory response [34]. Consistent with the previous studies [35], we successfully established SAKI rat model by applying the CLP surgery, which caused tubular apoptosis, aggravated systemic inflammatory response, and inflammatory cell infiltration in renal tissues. To reduce the impact on inflammatory response, antibiotics recommended in the guidelines [36] were not used in the present study when establishing the model. Some previous studies showed that activation of α7nAChR by PNU-282987 could ameliorate a variety of diseases, such as acute lung injury and intestinal epithelial dysfunction induced by sepsis [21,37]. Similarly, in this study, we observed that PNU-282987 reduced the levels of systemic inflammatory factors (TNF-α, IL-6, etc.), inflammatory cell infiltration and tubular apoptosis in renal tissues, thereby ameliorating renal histopathological injury. Moreover, PNU-282987 reduced the levels of AKI markers, that is, NGAL, KIM-1, Scr, and BUN, and increased urine volume in the CLP rats. These results suggest that PNU-282987 is effective in ameliorating SAKI by down-regulating the inflammatory response.

Tregs are a type of immune-suppressing cells [38]. Many published studies showed that adoptive transfer of Tregs has a protective effect on the kidneys of ischemia-reperfusion animals [13,39]. However, it is less documented in sepsis-related kidney injury. In this study, we proved that the infusion of Tregs could significantly improve the renal injury caused by SAKI in CLP rats. Lee et al. depleted Treg cells 96 hours before CLP surgery in mice and found that the kidney damage in mice was relieved 24 hours after surgery, which is contrary to our observation [40]. This might be explained by the injury severity of the model and other unknown changes after Tregs, which are yet to be verified. In the present study, we showed that the expression levels of CTLA-4 and Foxp3 in Tregs were increased in the CLP rats regardless of treatment by low or high doses of PNU-282987, suggesting that PNU-282987 can activate Tregs but may not be dose-sensitive which requires further study. IL-10 was not detected in the culture supernatant of Tregs in the presence or absence of PNU-282987 stimulation, which was consistent with results from Shevach et al. [41]. Furthermore, we found that the levels of TGF-β1 produced by Tregs did not change significantly on PNU-282987 treatment either *in vivo* or *in vitro*. These findings may suggest that IL-10 and TGF-β1 are not involved in PNU-282987-induced activation of Tregs. The α7nAChR, a ligand-gated ion channel, is widely expressed in a variety of cell types, including endothelial cells, monocytes, macrophages, and neutrophils [42]. The expression of α7nAChR in Tregs was demonstrated using reverse transcription-polymerase chain reaction, Western blot methods, and fluorescence labeling [43]. In our study, we confirmed the expression of α7nAChR in Tregs using similar methods. We further showed that the expression levels of CTLA-4 and Foxp3 in Tregs were significantly increased when Tregs were treated with PNU-282987. Moreover, adoptive transfer of isolated Tregs cocultured with low or high concentrations of PNU-282987 ameliorated AKI (Figure 8A-F) in the CLP rats. These results further demonstrated that the activation of Tregs by PNU-282987 can upregulate the inhibitory functions of Tregs which may, in turn, ameliorate renal injury, consistent with the previous studies showing that α7nAChR agonist, nicotine, could enhance Tregs inhibitory activity through α7nAChR activation [23]. Therefore, Tregs may be an immunotherapeutic target for alleviating SAKI, which worth further exploration. Moreover, the mechanism of PNU-282987 in regulating Treg cells to alleviate SAKI also requires further study, which might be promising in the clinical treatment of AKI.

## CONCLUSION

We showed at the molecular, cellular, and tissue level that PNU-282987 could activate Tregs. Moreover, PNU-282987-induced activation of Tregs can ameliorate SAKI in CLP rats. However, no significant difference was observed between the high and low doses of PNU-282987 groups, which might be due to a narrow difference in doses between the two groups. Further studies are needed to evaluate the effects of different doses of PNU-282987 on SAKI in CLP rats.

## Figures and Tables

**TABLE S1:**
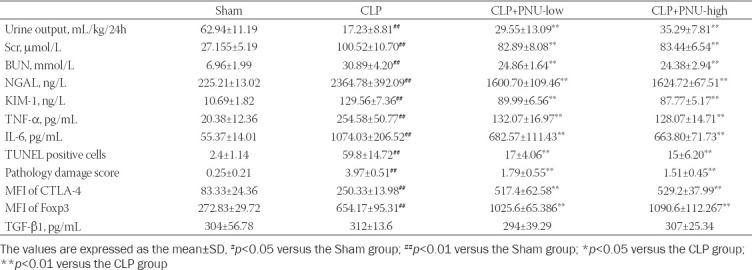
The variables related kidney, systemic inflammatory indicators, pathology damage score, and the MFI of CTLA-4 and Foxp3 after the injection of PNU-282987 in the SAKI rats

**TABLE S2:**
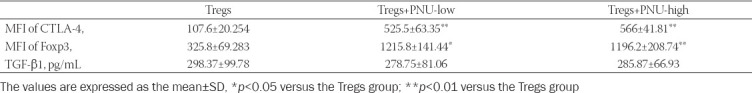
The MFI of CTLA-4 and Foxp3 and the production of TGF-β1 after coculture of isolated Tregs with different media

**TABLE S3:**
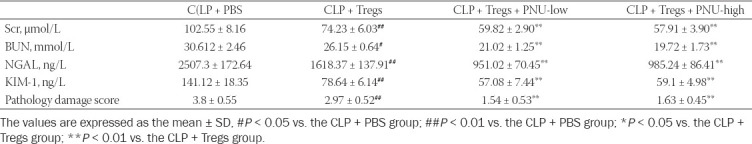
The biomarkers of early acute kidney injury and pathology damage score after the adoptive transfer of Tregs in the SAKI rats.

## References

[1] Sunggoro AJ, Arifin A, Marwanta S, Atmodjo SM, Maryono S (2016). 536P Characteristics and outcome of cancer patients with sepsis requiring admission to the intensive care unit. Ann Oncol.

[2] See EJ, Jayasinghe K, Glassford N, Bailey M, Johnson DW, Polkinghorne KR (2019). Long-term risk of adverse outcomes after acute kidney injury:A systematic review and meta-analysis of cohort studies using consensus definitions of exposure. Kidney Int.

[3] Bellomo R, Kellum JA, Ronco C, Wald R, Martensson J, Maiden M (2017). Acute kidney injury in sepsis. Intensive Care Med.

[4] van der Slikke EC, Star BS, van Meurs M, Henning RH, Moser J, Bouma HR (2021). Sepsis is associated with mitochondrial DNA damage and a reduced mitochondrial mass in the kidney of patients with sepsis-AKI. Crit Care.

[5] Gómez H, Kellum JA (2016). Sepsis-induced acute kidney injury. Curr Opin Crit Care.

[6] Bravo-Merodio L, Acharjee A, Hazeldine J, Bentley C, Foster M, Gkoutos GV (2019). Machine learning for the detection of early immunological markers as predictors of multi-organ dysfunction. Sci Data.

[7] Li X, Zou Y, Xing J, Fu YY, Wang K Y, Wan PZ (2020). Pretreatment with roxadustat (FG-4592) attenuates folic acid-induced kidney injury through antiferroptosis via Akt/GSK-3b/Nrf2 pathway. Oxid Med Cell Longev.

[8] Afzali B, Lombardi G, Lechler RI, Lord GM (2007). The role of T helper 17 (Th17) and regulatory T cells (Treg) in human organ transplantation and autoimmune disease. Clin Exp Immunol.

[9] Zhou S, Wu W, Wang Z, Wang Z, Su Q, Li X (2020). RelB regulates the homeostatic proliferation but not the function of Tregs. BMC Immunol.

[10] Hirota K, Duarte JH, Veldhoen M, Hornsby E, Li Y, Cua DJ (2011). Fate mapping of IL-17-producing T cells in inflammatory responses. Nat Immunol.

[11] Ghali JR, Wang YM, Holdsworth SR, Kitching AR (2016). Regulatory T cells in immune-mediated renal disease. Nephrology.

[12] Cho WY, Choi HM, Lee SY, Kim MG, Kim HK, Jo SK (2010). The role of Tregs and CD11c^+^ macrophages/dendritic cells in ischemic preconditioning of the kidney. Kidney Int.

[13] Kinsey GR, Huang L, Vergis AL, Li L, Okusa MD (2010). Regulatory T cells contribute to the protective effect of ischemic preconditioning in the kidney. Kidney Int.

[14] Liu M, Chien CC, Burne-Taney M, Molls RR, Racusen LC, Colvin RB (2016). A pathophysiologic role for T lymphocytes in murine acute cisplatin nephrotoxicity. J Am Soc Nephrol.

[15] Luan H, Wang C, Sun J, Zhao L, Li L, Zhou B (2020). Resolvin D1 protects against ischemia/reperfusion-induced acute kidney injury by increasing Treg percentages via the ALX/FPR2 pathway. Front Physiol.

[16] do Valle Duraes F, Lafont A, Beibel M, Martin K, Darribat K, Cuttat R (2020). Immune cell landscaping reveals a protective role for regulatory T cells during kidney injury and fibrosis. JCI Insight.

[17] Cho E, Lee JH, Lim HJ, Oh SW, Jo SK, Cho WY (2014). Soluble CD25 is increased in patients with sepsis-induced acute kidney injury. Nephrology.

[18] Tracey KJ (2019). Reflex control of immunity. Nat Rev Immunol.

[19] Skok MV, Grailhe R, Agenes F, Changeux JP (2007). The role of nicotinic receptors in B-lymphocyte development and activation. Life Sci.

[20] Inoue T, Abe C, Sun-Sang JS, Moscalu S, Jankowski J, Huang L (2016). Vagus nerve stimulation mediates protection from kidney ischemia-reperfusion injury through α7nAChR^+^ splenocytes. J Clin Invest.

[21] Pinheiro NM, Santana FP, Almeida RR, Guerreiro M, Martins MA, Caperuto LC (2017). Acute lung injury is reduced by the α7nAChR agonist PNU-282987 through changes in the macrophage profile. FASEB J.

[22] Gao Y, Kang K, Liu H, Kong W, Han Q, Zhang X (2017). GTS-21 attenuates LPS-induced renal injury via the cholinergic anti-inflammatory pathway in mice. Am J Transl Res.

[23] Wang DW, Zhou RB, Yao YM, Zhu XM, Yin YM, Zhao GJ (2010). Stimulation of α7 nicotinic acetylcholine receptor by nicotine increases suppressive capacity of naturally occurring CD4^+^ CD25^+^ regulatory T cells in mice *in vitro*. J Pharmacol Exp Ther.

[24] Luca C, Salvatore F, Vincenzo DP, Giovanni C, Attilio IL (2018). Anesthesia protocols in laboratory animals used for scientific purposes. Acta Biomed.

[25] Rittirsch D, Huber-Lang MS, Flierl MA, Ward PA (2019). Immunodesign of experimental sepsis by cecal ligation and puncture. Nat Protoc.

[26] Pinheiro NM, Miranda CJ, Santana FR, Bittencourt-Mernak M, Arantes-Costa FM, Olivo C (2020). Effects of VAChT reduction and α7nAChR stimulation by PNU-282987 in lung inflammation in a model of chronic allergic airway inflammation. Eur J Pharmacol.

[27] Rafieian-Kopaei M, Nasri H, Nematbakhsh M, Baradaran A, Gheissari A, Rouhi H (2012). Erythropoietin ameliorates genetamicin-induced renal toxicity:A biochemical and histopathological study. J Nephropathol.

[28] Paller MS, Hoidal JR, Ferris TF (1984). Oxygen free radicals in ischemic acute renal failure in the rat. J Clin Invest.

[29] Sheikh-Hamad D, Cacini W, Buckley AR, Isaac J, Truong LD, Tsao CC (2004). Cellular and molecular studies on cisplatin-induced apoptotic cell death in rat kidney. Arch Toxicol.

[30] Solanki MH, Chatterjee PK, Gupta M, Xue X, Plagov A, Metz MH (2014). Magnesium protects against cisplatin-induced acute kidney injury by regulating platinum accumulation. Am J Physiol Renal Physiol.

[31] Zheng C, Zhou Y, Huang Y, Chen B, Wu M, Xie Y (2019). Effect of ATM on inflammatory response and autophagy in renal tubular epithelial cells in LPS-induced septic AKI. Exp Ther Med.

[32] Chatterjee PK, Yeboah MM, Solanki MH, Kumar G, Xue X, Pavlov VA (2017). Activation of the cholinergic anti-inflammatory pathway by GTS-21 attenuates cisplatin-induced acute kidney injury in mice. PLoS One.

[33] Dejager L, Pinheiro I, Dejonckheere E, Libert, C (2011). Cecal ligation and puncture:The gold standard model for polymicrobial sepsis?. Trends Microbiol.

[34] Shi C, Wang Y, Chen Q, Jiao FZ, Pei MH, Gong ZJ (2020). Extracellular histone H3 induces pyroptosis during sepsis and may act through NOD2 and VSIG4/NLRP3 pathways. Front Cell Infect Microbiol.

[35] Zhao W, Zhang L, Chen R, Lu H, Sui M, Zhu Y (2018). SIRT3 protects against acute kidney injury via AMPK/mTOR-regulated autophagy. Front Physiol.

[36] Osuchowski MF, Ayala A, Bahrami S, Bauer M, Boros M, Cavaillon JM (2018). Minimum quality threshold in pre-clinical sepsis studies (MQTiPSS):An international expert consensus initiative for improvement of animal modeling in sepsis. Intensive Care Med Exp.

[37] Zhang Y, Zhou F, Wang Z, Li Z, Li J (2020). PNU-282987 attenuates intestinal epithelial barrier dysfunction in LPS-induced endotoxemia. Inflammation.

[38] Wing K, Sakaguchi S (2010). Regulatory T cells exert checks and balances on self tolerance and autoimmunity. Nat Immunol.

[39] Gandolfo MT, Jang HR, Bagnasco SM, Ko GJ, Agreda P, Satpute SR (2009). Foxp3+regulatory T cells participate in repair of ischemic acute kidney injury. Kidney Int.

[40] Lee SY, Lee YS, Choi HM, Ko YS, Lee HY, Jo SK (2012). Distinct pathophysiologic mechanisms of septic acute kidney injury:Role of immune suppression and renal tubular cell apoptosis in murine model of septic acute kidney injury. Crit Care Med.

[41] Shevach EM (2009). Mechanisms of foxp3+T regulatory cell-mediated suppression. Immunity.

[42] Wang H, Liao H, Ochani M, Justiniani M, Lin X, Yang L (2004). Cholinergic agonists inhibit HMGB1 release and improve survival in experimental sepsis. Nat Med.

[43] Marinou M, Tzartos SJ (2003). Identification of regions involved in the binding of alpha-bungarotoxin to the human alphα7 neuronal nicotinic acetylcholine receptor using synthetic peptides. Biochem J.

